# Supplementary material for: FtsK is Critical for the Assembly of the Unique Divisome Complex of the FtsZ-less Chlamydia trachomatis
Source: bioRxiv. 2025 Jan 9:2024.10.24.620021. Originally published 2024 Oct 24. Preprint. [Version 2] doi: 10.1101/2024.10.24.620021 (PMC11527202; doi:10.1101/2024.10.24.620021)
Supplement: 1 — Supp. Fig. S1:(A) Lysates were prepared from uninfected HeLa cells and HeLa cells infected with Ct L2. At 21hpi, lysates were prepared and characterized by immunoblotting with FtsK-specific antibodies. (B) HeLa cells were infected with Ct transformed with the various mCherry fusions used in this study; FtsK-mCherry (molecular mass - 114,664 Da), mCherry-PBP2 (molecular mass - 150,842 Da), mCherry-PBP3 (molecular mass - 100,150 Da), or mCherry-MreC (molecular mass - 63,906 Da). The fusions were induced with 10nM aTc at 17hpi. HeLa cells were harvested at 21hpi and lysates were prepared and characterized by immunoblotting analysis with a rabbit polyclonal mCherry antibodies. (C) HeLa cells were infected with Ct transformed N-terminal fusions of PBP2 or PBP3. The fusions were induced (+aTc) at17hpi. Controls were not induced (−aTc). The cells were harvested at 21hpi and lysates were prepared as described in the Methods and characterized by immunoblotting analysis with rabbit antibodies raised against peptides derived from chlamydial PBP2 or PBP3. The PBP3 antibody primarily detects a single species with the predicted molecular mass of mCherry-PBP3 in the induced sample. The PBP2 antibody primarily detects a species of ~120kD in the induced sample, which is smaller than the predicted molecular mass of mCherry-PBP2 (~150kD). The failure to detect full length mCherry-PBP2 may be due to the masking of the epitope recognized by the PBP2 antibody by the N-terminal mCherry tag in the full-length protein. (D) HeLa cells were infected with Ct transformed with mCherry-PBP2 or mCherry-PBP3. The fusions were induced by the addition of 10nM aTc to the media of the infected cells at 19hpi. Infected cells were harvested at 21hpi and lysates were prepared and stained with the PBP2 or PBP3 antibodies. The staining with the PBP2 and PBP3 antibodies completely overlaps the mCherry fluorescence from the mCherry-PBP2 and mCherry-PBP3 fusions (Bars are 3μm) Supp. Fig. S2: (A) HeLa cells wer [file NIHPP2024.10.24.620021V2-supplement-1.pdf]

bioRxiv preprint doi: https://doi.org/10.1101/2024.10.24.620021; this version posted January 9, 2025. The copyright holder for this preprint (which was not certified by peer review) is the author/funder, who has granted bioRxiv a license to display the preprint in perpetuity. It is made available under aCC-BY 4.0 International license.

Supp. Figure S1

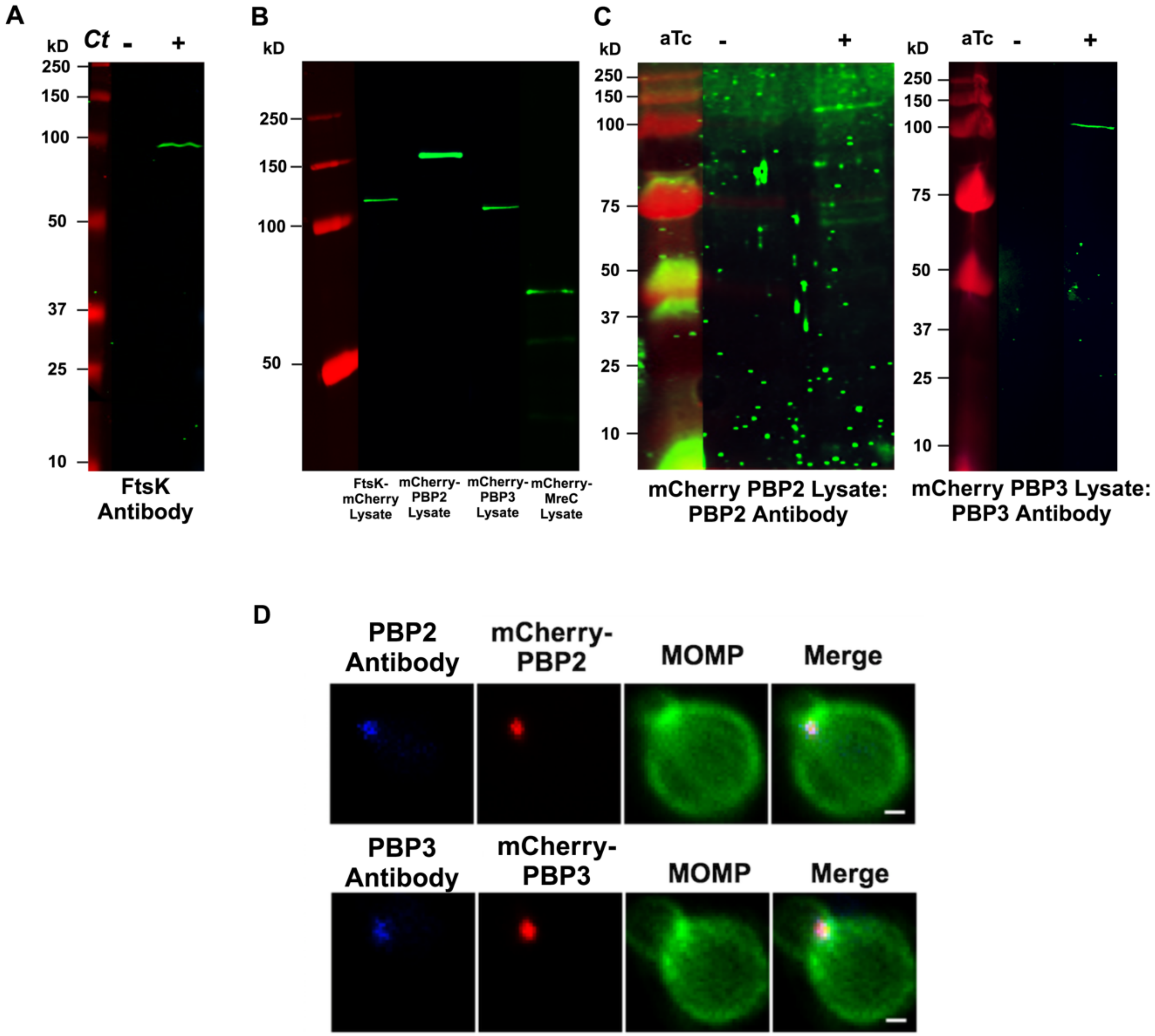

bioRxiv preprint doi: https://doi.org/10.1101/2024.10.24.620021; this version posted January 9, 2025. The copyright holder for this preprint (which was not certified by peer review) is the author/funder, who has granted bioRxiv a license to display the preprint in perpetuity. It is made available under aCC-BY 4.0 International license.

Supp. Figure S2

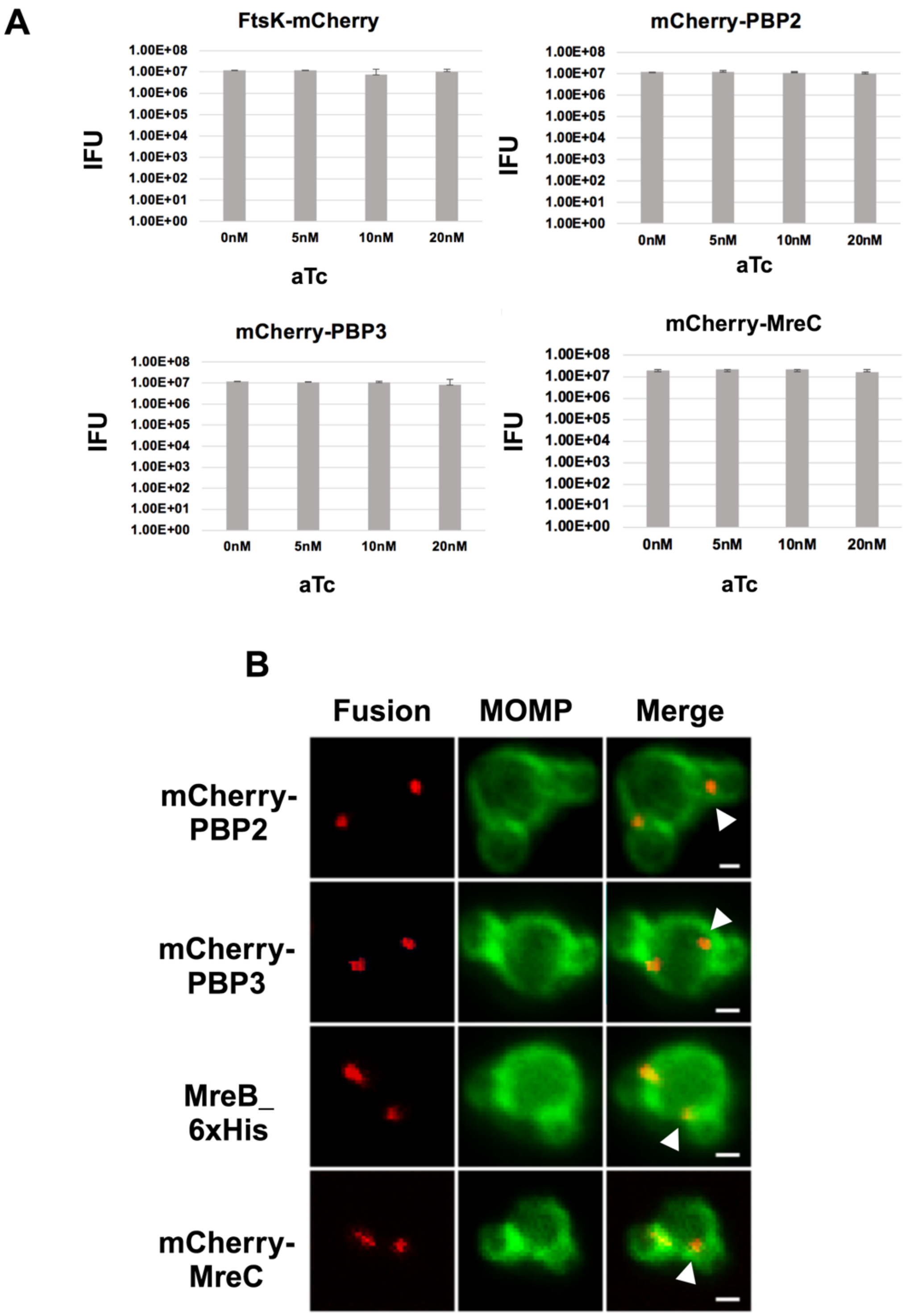

bioRxiv preprint doi: https://doi.org/10.1101/2024.10.24.620021; this version posted January 9, 2025. The copyright holder for this preprint (which was not certified by peer review) is the author/funder, who has granted bioRxiv a license to display the preprint in perpetuity. It is made available under aCC-BY 4.0 International license.

Supp. Figure S3

A

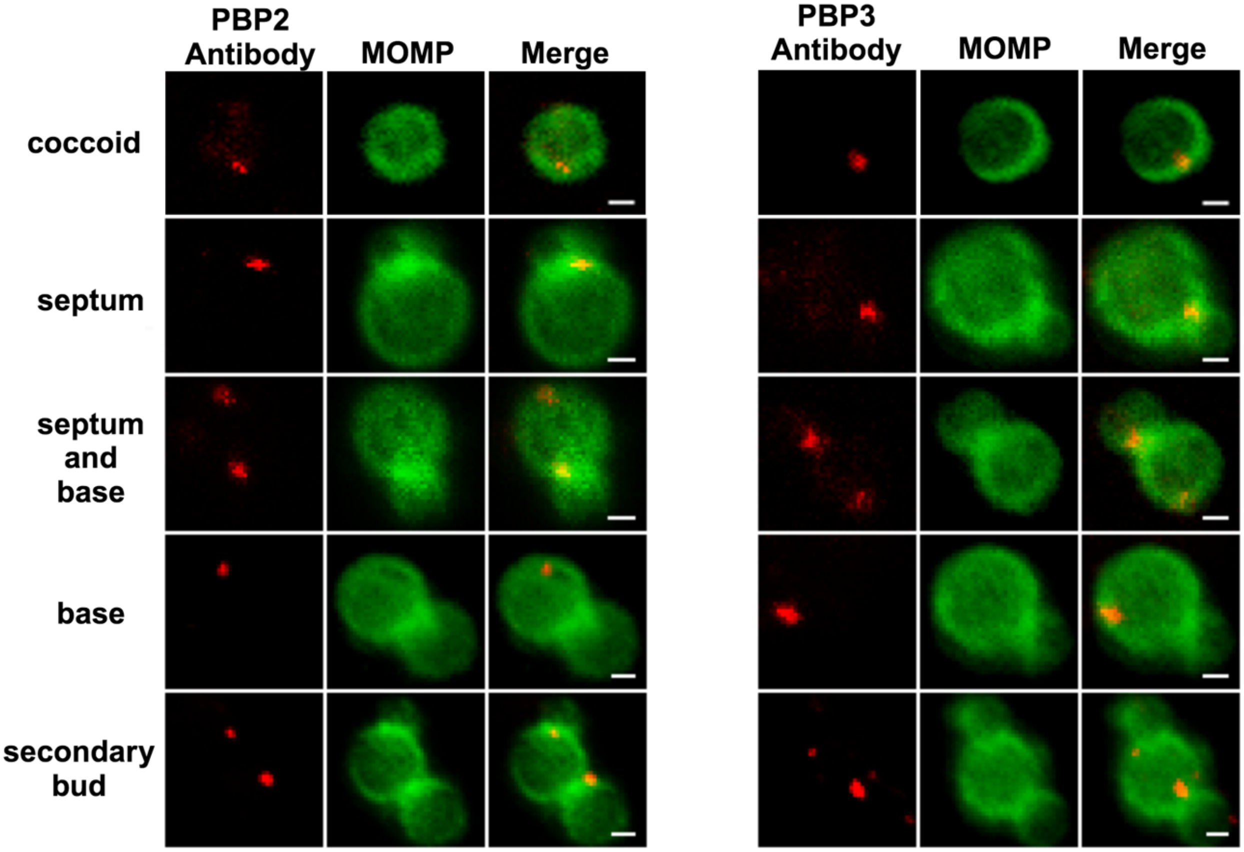

B

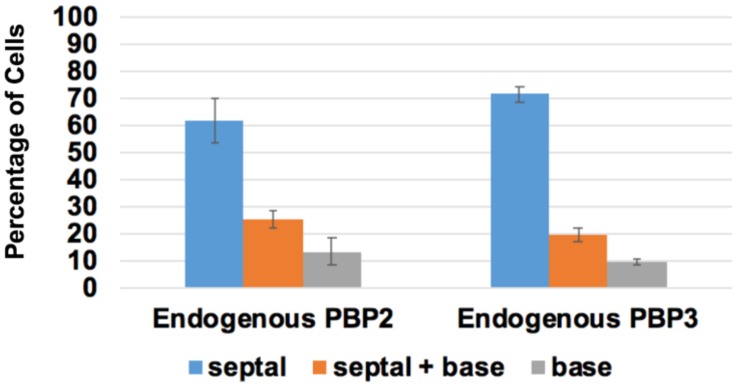

bioRxiv preprint doi: https://doi.org/10.1101/2024.10.24.620021; this version posted January 9, 2025. The copyright holder for this preprint (which was not certified by peer review) is the author/funder, who has granted bioRxiv a license to display the preprint in perpetuity. It is made available under aCC-BY 4.0 International license.

Supp. Figure S4

A

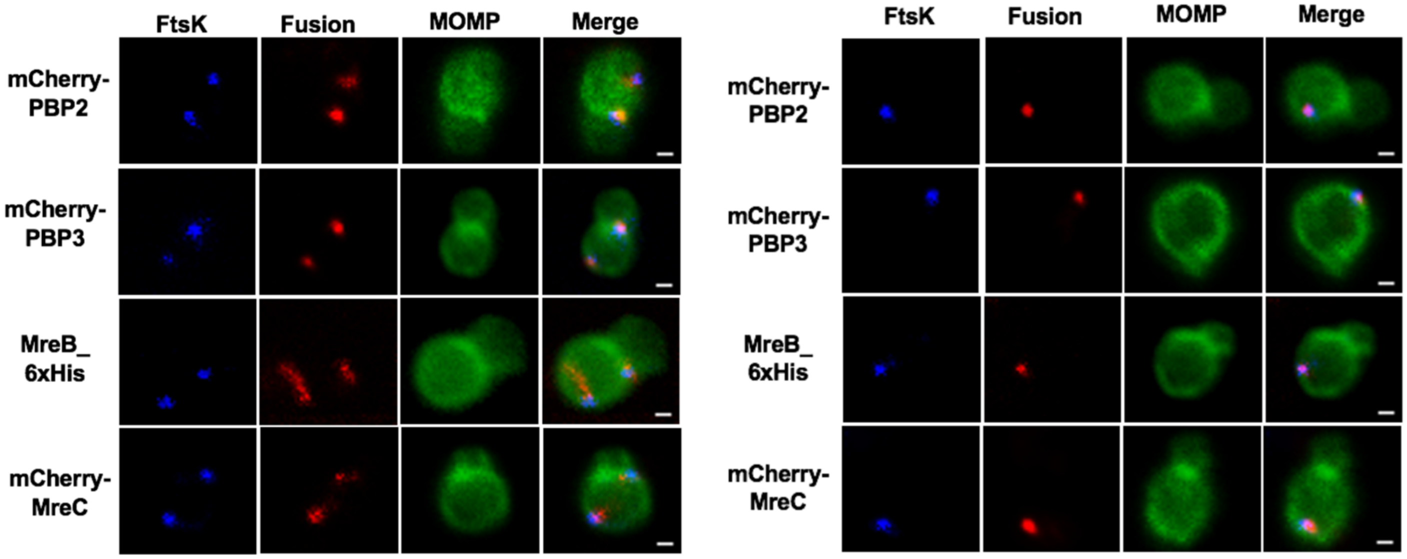

B

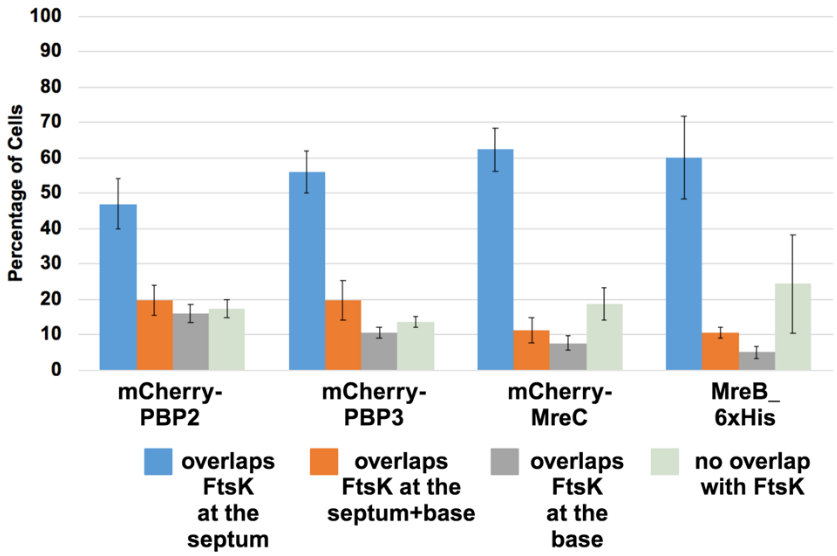

bioRxiv preprint doi: https://doi.org/10.1101/2024.10.24.620021; this version posted January 9, 2025. The copyright holder for this preprint (which was not certified by peer review) is the author/funder, who has granted bioRxiv a license to display the preprint in perpetuity. It is made available under aCC-BY 4.0 International license.

Supp. Figure S5

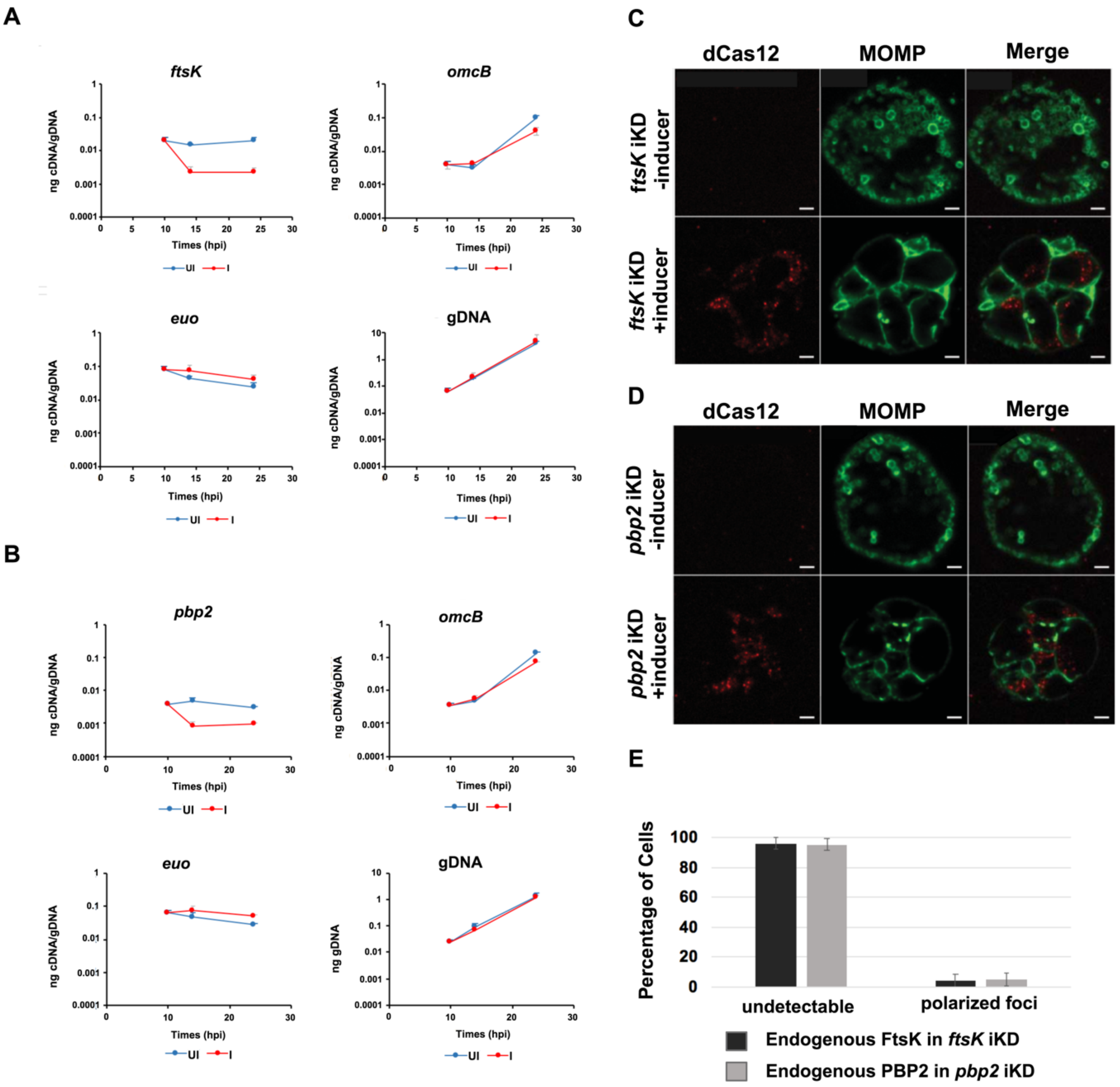

bioRxiv preprint doi: https://doi.org/10.1101/2024.10.24.620021; this version posted January 9, 2025. The copyright holder for this preprint (which was not certified by peer review) is the author/funder, who has granted bioRxiv a license to display the preprint in perpetuity. It is made available under aCC-BY 4.0 International license.

Supp. Figure S6

A

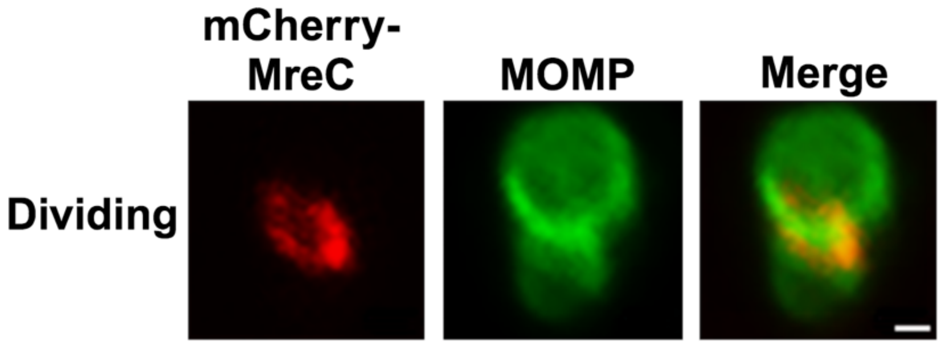

B

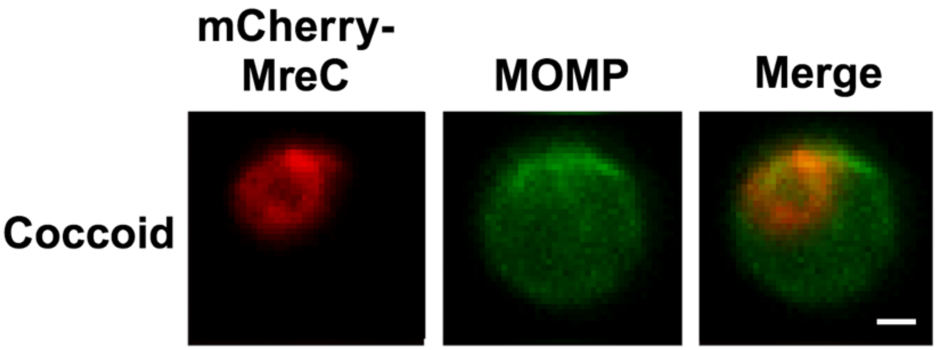

bioRxiv preprint doi: https://doi.org/10.1101/2024.10.24.620021; this version posted January 9, 2025. The copyright holder for this preprint (which was not certified by peer review) is the author/funder, who has granted bioRxiv a license to display the preprint in perpetuity. It is made available under aCC-BY 4.0 International license.

Supp. Table S1

Primer Name	Primer Sequence (5'-3')	GC content (%)	TM (°C)
FtsK_mCherry (fwd)	AGAGGAGAAAGGATCTGCGGCCGCATGGGAAAAGAACGGAAGAAAGCAAG	52	72.8
FtsK_mCherry (rvs)	CCCTTAGAGACCATTGCGGCCGCATCGTCCTGATTTGATAATTGGACTAGTATTTGAC	47	72.4
mCherry_PBP2 (fwd)	ATGGTCTCTAAGGGCGAGGAAGAC	54	59.1
mCherry_PBP2 (rvs)	ATGGTCGACCGGTACCTTAGCTGAAAGATTTTTTACGAATCTCTTCCCATTTCTCTATC	41	70.2
mCherry_PBP3 (fwd)	ATGGTCTCTAAGGGCGAGGAAGAC	54	59.1
mCherry_PBP3 (rvs)	ATGGTCGACCGGTACCCTATTTGCGATTCCATTCCTCATATAGCAG	48	69.9
mCherry_MreC (fwd)	ATGGTCTCTAAGGGCGAGGAAGAC	54	59.1
mCherry_MreC (rvs)	ATGGTCGACCGGTACCCTACTCCCAAATCAAACCAAAAATATCAGGACG	47	70.4
Plasmid	pBOMB4-tet		

bioRxiv preprint doi: https://doi.org/10.1101/2024.10.24.620021; this version posted January 9, 2025. The copyright holder for this preprint (which was not certified by peer review) is the author/funder, who has granted bioRxiv a license to display the preprint in perpetuity. It is made available under aCC-BY 4.0 International license.

Supp. Table S2

Primer Name	Primer Sequence (5'-3')	GC content (%)	TM (°C)
ftsK (fwd)	CGACTCCAAGTTCCTCTTCTTC	39.1	55.2
ftsK (rvs)	GATCCAGTGGTTCCTGCAATA	47.6	54.6
pbp2 (fwd)	TAACACTGACGCGGAACATAG	47.6	55.1
pbp2 (rvs)	CCGAAAGCATGAGCAGATAGA	47.6	54.8
omcB (fwd)	CGGTAGGATCTCCCTATCCTATT	47.8	54.4
omcB (rvs)	CGAACTCTGCTTCACATGGTA	47.6	55
euo (fwd)	CGAAGACTACTCGTTGGGAAATA	43.5	54.7
euo (rvs)	AACAGAAGCTCTCCTTGATAAGT	39.1	53.8
