# Supplementary material for: FtsK Initiates the Assembly of a Unique Divisome Complex in the FtsZ-less Chlamydia trachomatis
Source: bioRxiv. 2024 Oct 24:2024.10.24.620021. Preprint. [Version 1] doi: 10.1101/2024.10.24.620021 (PMC11527202; doi:10.1101/2024.10.24.620021)
Supplement: Supplementary file 1 [file NIHPP2024.10.24.620021v1-supplement-1.pdf]

bioRxiv preprint doi: https://doi.org/10.1101/2024.10.24.620021; this version posted October 24, 2024. The copyright holder for this preprint (which was not certified by peer review) is the author/funder, who has granted bioRxiv a license to display the preprint in perpetuity. It is made available under aCC-BY 4.0 International license.

Supplemental Figure 1

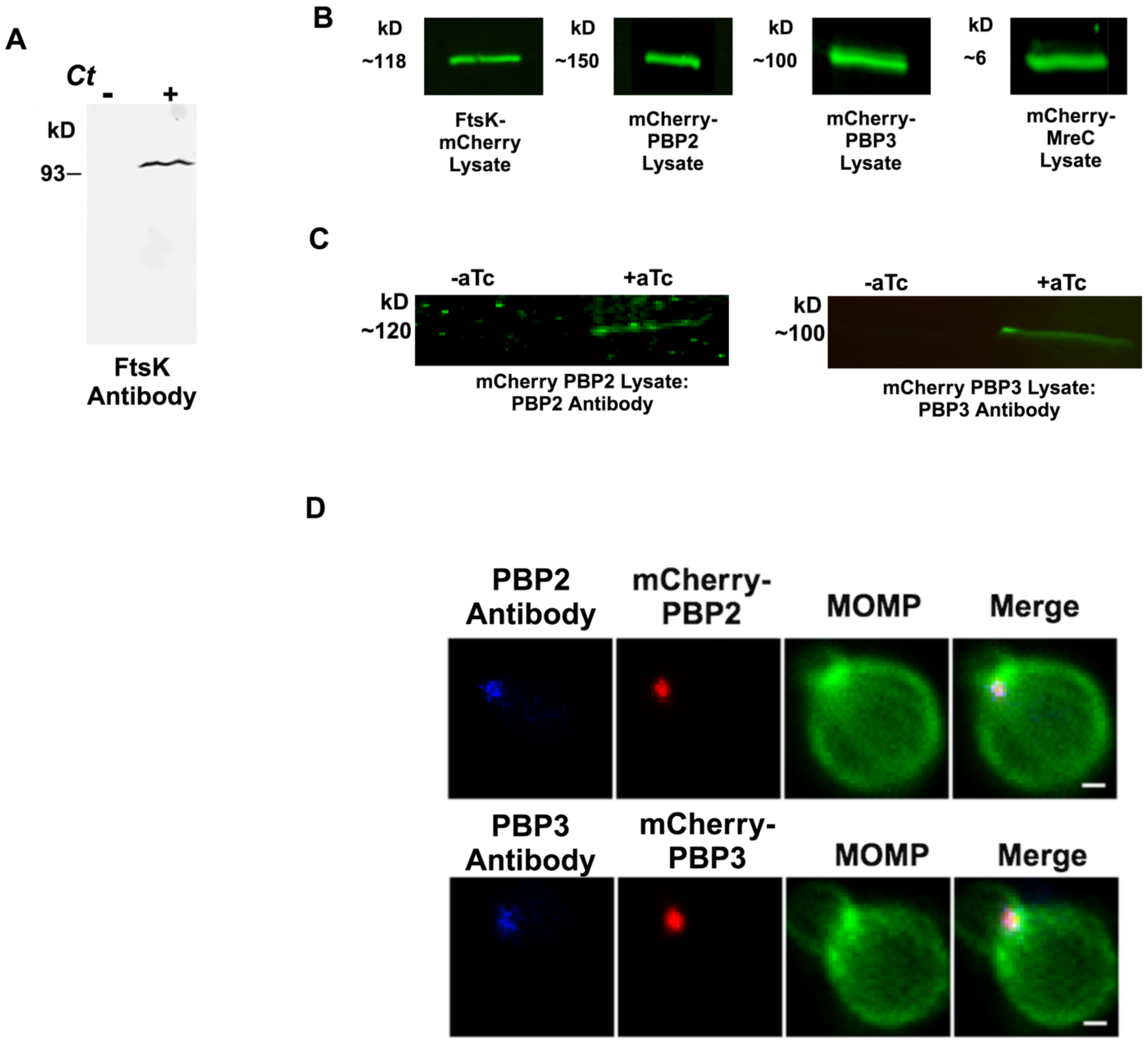

bioRxiv preprint doi: https://doi.org/10.1101/2024.10.24.620021; this version posted October 24, 2024. The copyright holder for this preprint (which was not certified by peer review) is the author/funder, who has granted bioRxiv a license to display the preprint in perpetuity. It is made available under aCC-BY 4.0 International license.

Supplemental Figure 2

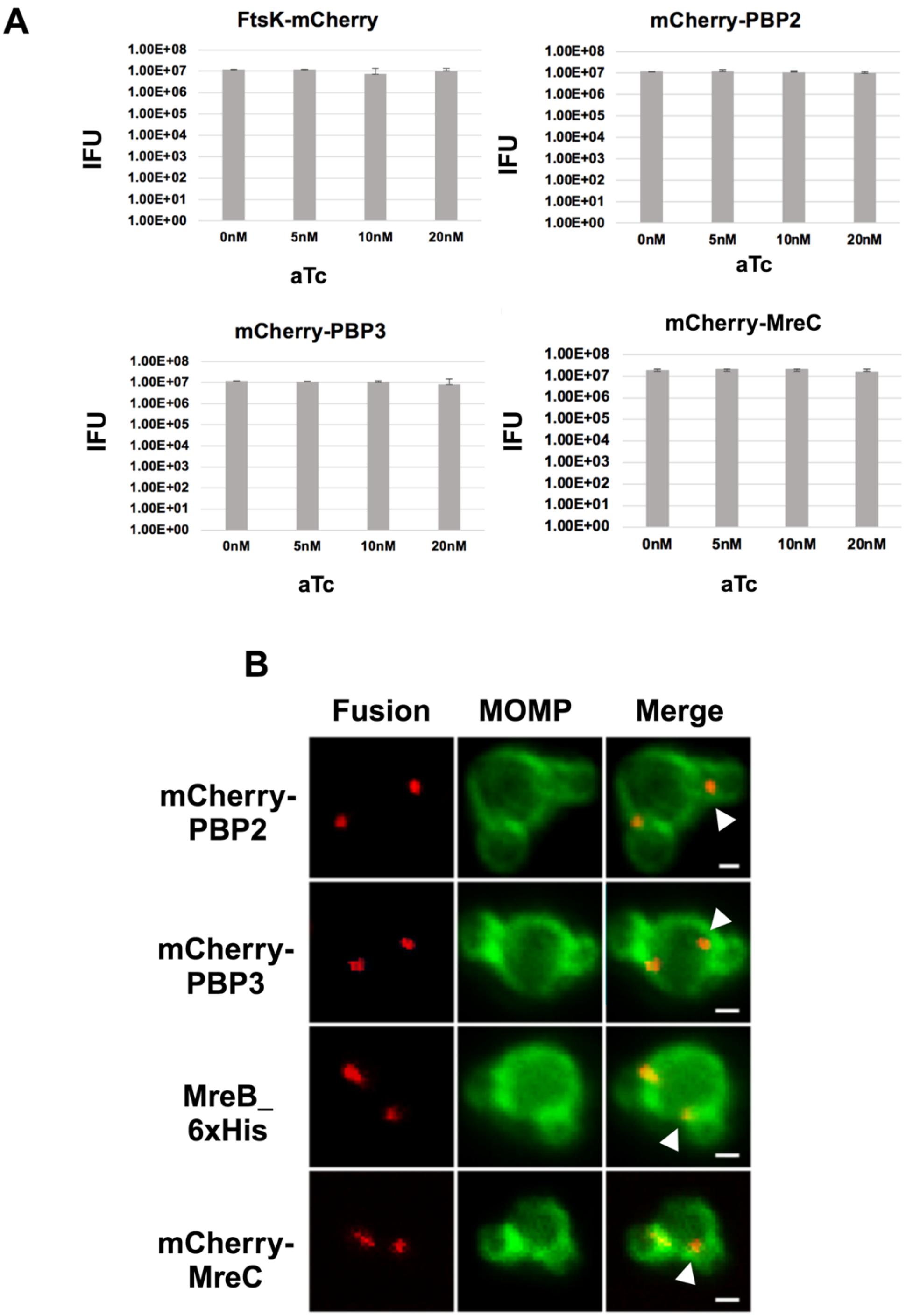

bioRxiv preprint doi: https://doi.org/10.1101/2024.10.24.620021; this version posted October 24, 2024. The copyright holder for this preprint (which was not certified by peer review) is the author/funder, who has granted bioRxiv a license to display the preprint in perpetuity. It is made available under aCC-BY 4.0 International license.

838 **Supplemental Figure 3**

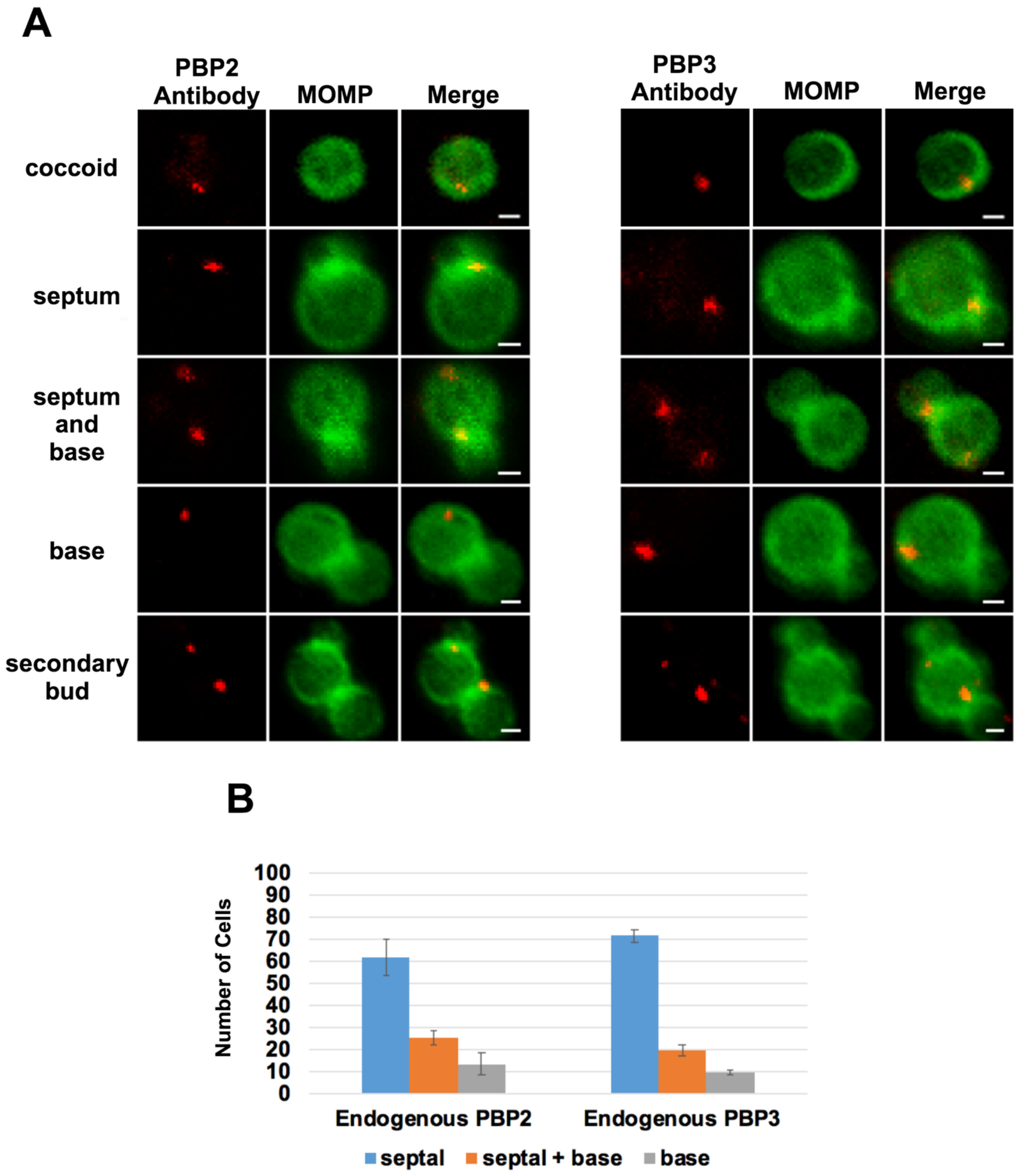

bioRxiv preprint doi: https://doi.org/10.1101/2024.10.24.620021; this version posted October 24, 2024. The copyright holder for this preprint (which was not certified by peer review) is the author/funder, who has granted bioRxiv a license to display the preprint in perpetuity. It is made available under aCC-BY 4.0 International license.

Supplemental Figure 4

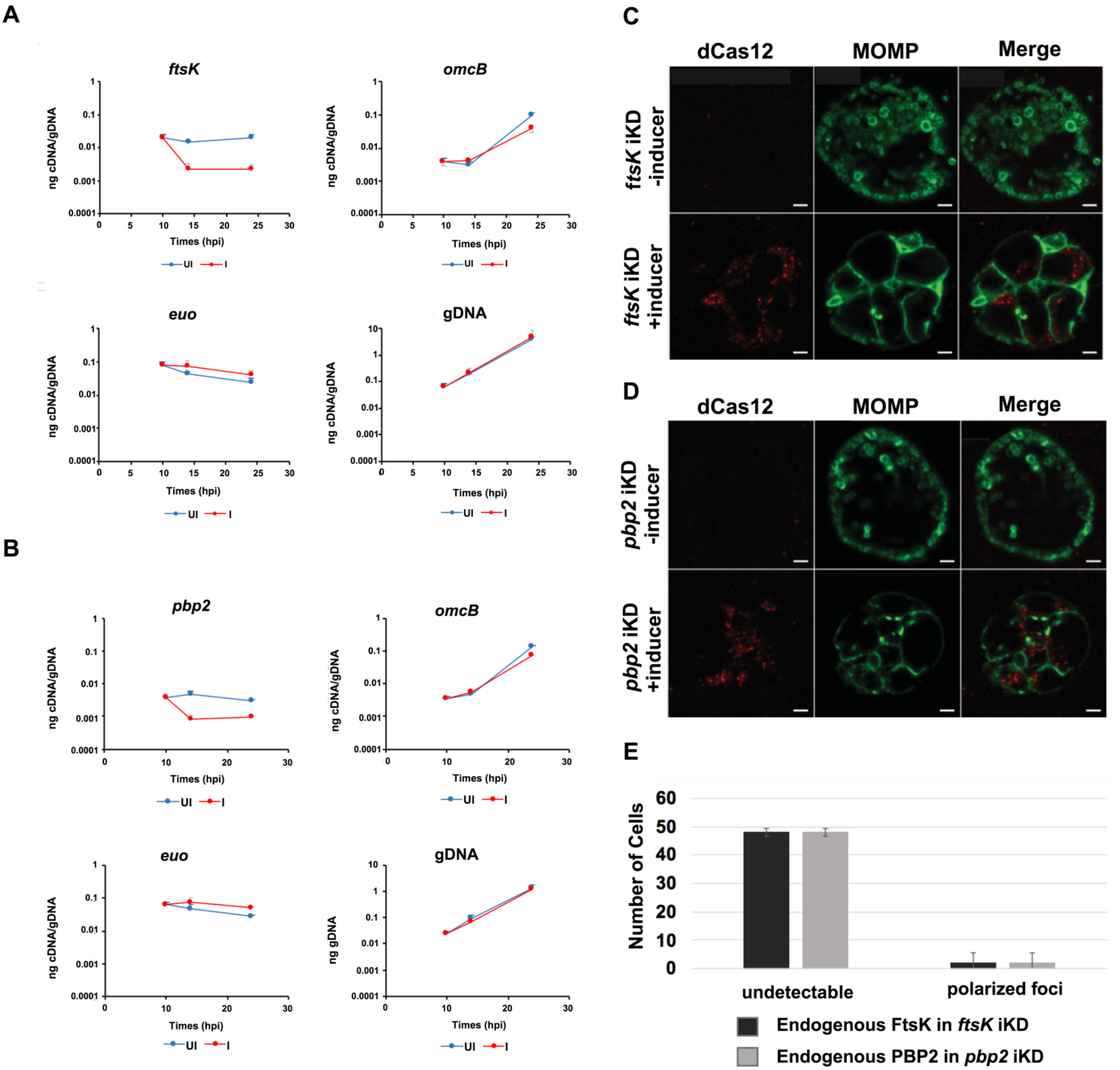

bioRxiv preprint doi: https://doi.org/10.1101/2024.10.24.620021; this version posted October 24, 2024. The copyright holder for this preprint (which was not certified by peer review) is the author/funder, who has granted bioRxiv a license to display the preprint in perpetuity. It is made available under aCC-BY 4.0 International license.

Supplemental Figure 5

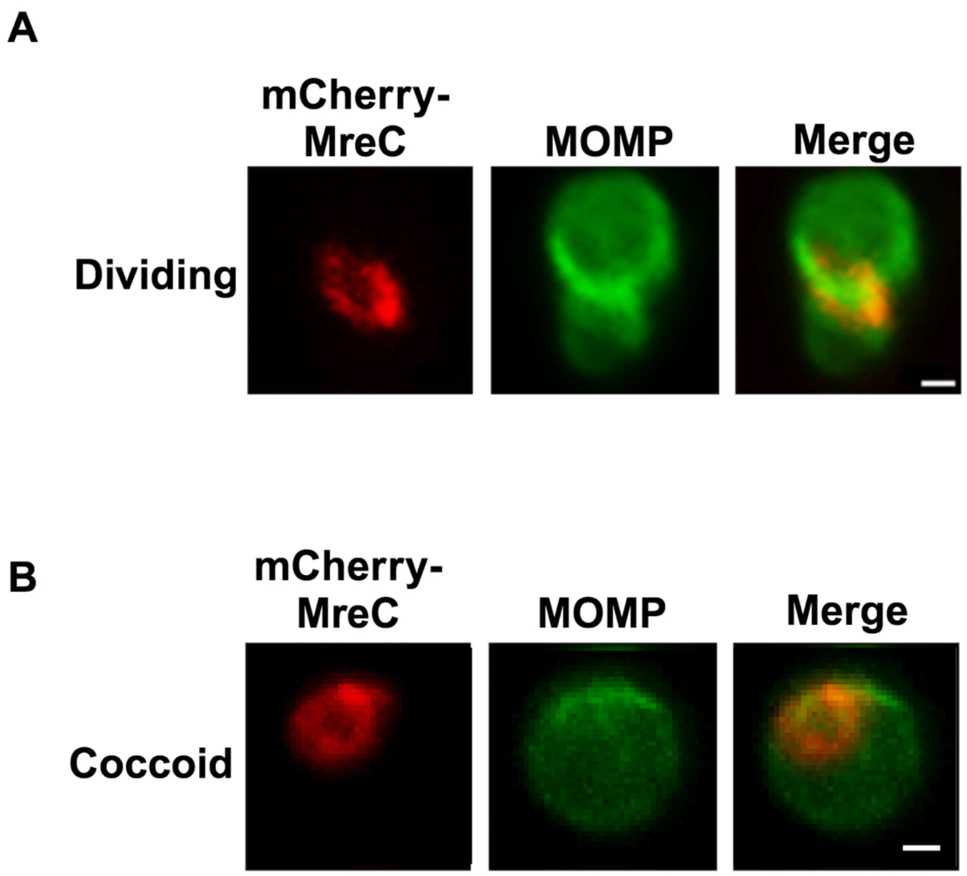

bioRxiv preprint doi: https://doi.org/10.1101/2024.10.24.620021; this version posted October 24, 2024. The copyright holder for this preprint (which was not certified by peer review) is the author/funder, who has granted bioRxiv a license to display the preprint in perpetuity. It is made available under aCC-BY 4.0 International license.

Supplemental Table 1

Primer Name	Primer Sequence (5'-3')	GC content (%)	TM (°C)
FtsK_mCherry (fwd)	AGAGGAGAAAGGATCTGCGGCCGCATGGGAAAAGAACGGAAGAAAGCAAG	52	72.8
FtsK_mCherry (rvs)	CCCTTAGAGACCATTGCGGCCGCATCGTCCTGATTTGATAATTGGACTAGTATTTGAC	47	72.4
mCherry_PBP2 (fwd)	ATGGTCTCTAAGGGCGAGGAAGAC	54	59.1
mCherry_PBP2 (rvs)	ATGGTCGACCGGTACCTTAGCTGAAAGATTTTTTACGAATCTCTTCCCATTTCTCTATC	41	70.2
mCherry_PBP3 (fwd)	ATGGTCTCTAAGGGCGAGGAAGAC	54	59.1
mCherry_PBP3 (rvs)	ATGGTCGACCGGTACCCTATTTGCGATTCCATTCCTCATATAGCAG	48	69.9
mCherry_MreC (fwd)	ATGGTCTCTAAGGGCGAGGAAGAC	54	59.1
mCherry_MreC (rvs)	ATGGTCGACCGGTACCCTACTCCCAAATCAAACCAAAAATATCAGGACG	47	70.4
Plasmid	pBOMB4-tet		

bioRxiv preprint doi: https://doi.org/10.1101/2024.10.24.620021; this version posted October 24, 2024. The copyright holder for this preprint (which was not certified by peer review) is the author/funder, who has granted bioRxiv a license to display the preprint in perpetuity. It is made available under aCC-BY 4.0 International license.

Supplemental Table 2

Primer Name	Primer Sequence (5'-3')	GC content (%)	TM (°C)
ftsK (fwd)	CGACTCCAAGTTCCTCTTCTTC	39.1	55.2
ftsK (rvs)	GATCCAGTGGTTCCTGCAATA	47.6	54.6
pbp2 (fwd)	TAACACTGACGCGGAACATAG	47.6	55.1
pbp2 (rvs)	CCGAAAGCATGAGCAGATAGA	47.6	54.8
omcB (fwd)	CGGTAGGATCTCCCTATCCTATT	47.8	54.4
omcB (rvs)	CGAACTCTGCTTCACATGGTA	47.6	55
euo (fwd)	CGAAGACTACTCGTTGGGAAATA	43.5	54.7
euo (rvs)	AACAGAAGCTCTCCTTGATAAGT	39.1	53.8
